# The Utility and Implications of Ambient Scribes in Primary Care

**DOI:** 10.2196/57673

**Published:** 2024-10-04

**Authors:** Puneet Seth, Romina Carretas, Frank Rudzicz

**Affiliations:** 1 Department of Family Medicine McMaster University Hamilton, ON Canada; 2 School of Public Health University of Alberta Edmonton, AB Canada; 3 Faculty of Computer Science Dalhousie University Halifax, NS Canada; 4 Vector Institute for Artificial Intelligence Toronto, ON Canada

**Keywords:** artificial intelligence, AI, large language model, LLM, digital scribe, ambient scribe, organizational efficiency, electronic health record, documentation burden, administrative burden

## Abstract

Ambient scribe technology, utilizing large language models, represents an opportunity for addressing several current pain points in the delivery of primary care. We explore the evolution of ambient scribes and their current use in primary care. We discuss the suitability of primary care for ambient scribe integration, considering the varied nature of patient presentations and the emphasis on comprehensive care. We also propose the stages of maturation in the use of ambient scribes in primary care and their impact on care delivery. Finally, we call for focused research on safety, bias, patient impact, and privacy in ambient scribe technology, emphasizing the need for early training and education of health care providers in artificial intelligence and digital health tools.

## Introduction

Integrating artificial intelligence (AI) in health care has opened new horizons for improving clinical efficiency and patient care. Given the integral role that communication plays in all aspects of clinical care, particularly during patient-physician conversation, using AI to enhance communication and reduce workflow friction has immense implications. Ambient scribes are AI-powered systems that passively listen to and analyze health care provider–patient conversations, automatically generating accurate clinical documentation. Leveraging automatic speech recognition and modern forms of AI, ambient scribes stand at the forefront of the health AI revolution [1].

Large language models (LLMs), a form of AI trained on massive amounts of data that can generate text and respond to requests as if they understand them, have been a recent catalyst in the capabilities of ambient scribes. Initially, automatic speech recognition demonstrated moderate accuracy in converting speech to text and lacked contextual understanding [2]. However, more modern neural network models such as ClinicalBERT [3], leveraging components based on transformer networks, offer more nuanced understanding and text generation nearly indistinguishable from human performance [4]. The internal mechanisms of these transformers, including self-attention components, may enable models to discern relevant parts of conversations, which is essential in complex health care dialogues [5]. Moreover, recent LLMs such as GPT-4, developed with reinforcement learning, have shown capabilities beyond traditional models, including passing scores on all steps of the USMLE (United States Medical Licensing Examination), demonstrating understanding and potential across medical contexts [4].

## Challenges in Primary Care

In contemporary health care, primary care is experiencing an acute strain, arguably more so than other medical disciplines [6]. The sector is grappling with significant challenges, most notably health care provider burnout and an escalating human resource crisis [6]. In Canada, 2023 marked an unprecedented trend with a record number of unfilled positions in primary care residency programs [7]. Concurrently, there has been an alarming increase in the number of primary care providers leaving the profession, a phenomenon partly attributable to the overwhelming administrative burdens they face [6]. Primary care, characterized by its multifaceted nature—commonly encompassing multi-issue visits, ambiguous clinical presentations, and a diverse array of visit types—demands significant administrative work from providers [8]. This, combined with the inherently unique characteristics of primary care consultations, positions this domain to benefit from the adoption of ambient scribes. By alleviating some of the administrative pressures, ambient scribes may significantly mitigate these pain points, offering hope for an overburdened primary care system.

While comprehensive data on ambient scribe use in health care is sparse, anecdotal evidence suggests a growing adoption in primary care [9]. These tools have shown potential in reducing the administrative burden, allowing clinicians to focus more on patient care. This shift is particularly evident in primary care, where the diversity and ambiguity of clinical presentations demands flexible and efficient documentation methods [10].

## The Stages of Maturation of Ambient Scribe Use in Primary Care

The advancement of ambient scribe utilization within primary care can be described in a staged process based on the nature of the activities that are supported by the tool. We posit four high-level stages, shown in Table 1. The rationale behind the four stages is based on an ascending degree of complexity associated with several factors, including technical complexity in development, medicolegal barriers to adoption, and cultural factors in the practice of medicine that would impact adoption [5,11].

**Table 1:** Key activities associated with various stages of ambient scribe maturation in a clinical setting.

Key activity	Stage 1	Stage 2	Stage 3	Stage 4
Automation of clinical documentation	✓	✓	✓	✓
Automation of administrative actions		✓	✓	✓
Reactive clinical decision support			✓	✓
Proactive clinical decision support				✓

Stage 1 describes the most basic ambient scribe functionality, in which the tools exclusively automate clinical documentation. This may involve integration with an electronic medical record (EMR) and typically does not require information retrieval from the EMR. Stage 2 adds the ability of the ambient scribe to address administrative workflow improvements for the clinician, such as generating a letter, filling out a form, or generating tasks to be completed. Most present-day ambient scribes are likely in stages 1 or 2.

Stage 3 introduces the first clinical decision support capabilities of the ambient scribe. These would be reactive, in that they would be initiated by the clinician. For example, the clinician could consult the ambient scribe with a clinical question, such as asking about the dosing of a medication or other diagnostic possibilities. This would necessitate that the ambient scribe has access to medical knowledge and has been trained for this purpose.

Lastly, stage 4, which we imagine to be achievable in the near future, would represent the ambient scribe playing a proactive clinical decision support role during the visit, thereby having the greatest extent of impact on the evolution of the clinical encounter. As an example, while a clinician is taking a history from the patient, if a relevant question is missed (for example, screening for hypertension or migraines in a patient being initiated on an oral contraceptive), the ambient scribe may proactively prompt the clinician through a visual cue to assist further history taking. Similarly, an advanced ambient scribe could alert the clinician and patient on other relevant issues to discuss that may not have been brought up during the visit but are time-sensitive (eg, a finding on a recent diagnostic imaging test that has not been addressed). In this way, it can be appreciated that the ambient scribe can serve as an important interface between the clinician, the patient, and an evolving series of computational enhancements that may be available.

## Barriers and Considerations

Several important considerations need to be addressed for the safe deployment of ambient scribes as they mature in capability and use. Several of these relate to AI in medicine in general [12]. Some general considerations include:

- The privacy of personal health information that may be collected by vendors of AI tools, raising concerns around data security, consent, and potential misuse of sensitive information
- Limited generalizability of these tools to populations beyond those with which they were tested or trained; the applicability of AI tools can vary across clinical settings and patient populations, as its performance in one context may not translate to another (eg, a tool optimized for primary care settings and focused on managing chronic conditions may not operate as effectively in specialized acute care settings like cardiology)
- The amplification of biases that may be inherent to the datasets in which these tools are trained; for example, if an AI model is trained on data that does not include patients from an appropriately diverse range of ethnicities and socioeconomic backgrounds, it may be biased or overfit to a limited population [5,13]

In addition, several other considerations exist in the use of ambient scribes. First, it is important to consider the unique impact that the recording of a patient-physician conversation may have on the therapeutic utility of the encounter. The patient-physician conversation is considered confidential, and its effectiveness is dependent on the patient feeling comfortable and free to disclose personal and intimate information [14]. There is limited literature at present investigating the patient’s perception of their visit being recorded by an ambient scribe. Furthermore, it is still being determined whether this may impact the nature of their responses during the visit. Assuming informed consent for the technology has taken place in which the value proposition of the technology is clearly explained, we hypothesize that patients will receive the use of this technology positively, as it should aid in reducing documentation strain on the physician, thus allowing them to be more focused on the human interaction. Second, it is well documented that new technology implementation in health care delivery often requires substantive change management, even when the benefits of the technology being implemented are well known [11]. While initially it may appear that there are no significant additional tasks necessary for the physician with ambient scribes, there may be net new tasks as well as appreciable losses in existing workflows. The physician (or another team member in the clinic) may be required to obtain consent from the patient to use the ambient scribe and answer questions about the technology. Additionally, it must be stressed that while the clinical visit may be documented automatically, the clinician must still review the output from the ambient scribe and correct any errors or omissions. Indeed, the accuracy of ambient scribes depends on various unique factors including diversity of linguistic backgrounds, microphone variability and audio quality (including exclusion of background sounds), changing and advanced medical terminologies, and challenges with context awareness in semistructured conversation. That is, identifying which parts of the conversation are pertinent to medical documentation is a unique challenge. Continuous learning involving both audio and language modeling will be necessary at the site level. How these AI operations may potentially involve third-party software vendors without violating privacy is also an open question. Given physicians may be leveraging other workflow optimization tools to aid with clinical documentation, such as clinical note EMR templates, they may experience an initial degradation of their workflow. Lastly, procedures should be put in place that specify whether whole conversations should be saved, whether only utterances from one party are necessary, and for how long recordings are to be retained (eg, for auditing or retraining).

As ambient scribe capabilities advance, as described in stages 3 and 4 above, the nature of the clinical encounter may be subject to inherent changes. Over time, ambient scribes and related AI technologies will likely play a greater role in clinical decision-making around clinical diagnosis and management of the patient. This includes active, real-time recommendations from the scribe, which must be managed by the physician. This will lead to an evolution in the role of the primary care physician, requiring them to have greater foundational knowledge on the use, benefits, and limitations of AI and allowing them to focus more on shared decision-making, empathetic communication, and therapeutic relationship development [15]. Modernization of medical training and family medicine residency curricula will be necessary to account for these changes and upskill the existing labor force.

## Conclusion

Ambient scribes, powered by LLMs, offer a promising avenue for enhancing clinical practice in primary care. Their ability to reduce administrative load, improve documentation accuracy, and potentially aid in clinical decision-making positions them as valuable assets in modern health care. However, their efficacy and safety must be validated through further research. The risk of amplifying harmful bias, the applicability and accuracy of their function in diverse primary care settings, and patient perception and change management, among other considerations, must be taken into account. Given the immense pressures that exist on primary care today, we must address these and reap the benefits of this powerful technology.

## Abbreviations

AI: artificial intelligence
EMR: electronic medical record
LLM: large language model
USMLE: United States Medical Licensing Examination
